# Acute exacerbations of COPD are associated with significant activation of matrix metalloproteinase 9 irrespectively of airway obstruction, emphysema and infection

**DOI:** 10.1186/s12931-015-0240-4

**Published:** 2015-06-28

**Authors:** Eleni Papakonstantinou, George Karakiulakis, Spyros Batzios, Spasenija Savic, Michael Roth, Michael Tamm, Daiana Stolz

**Affiliations:** Clinic of Pulmonary Medicine and Respiratory Cell Research, University Hospital Basel, Petersgraben 4, 4031 Basel, Switzerland; Department of Pharmacology, School of Medicine, Aristotle University of Thessaloniki, 54124 Thessaloniki, Greece; Institute for Pathology, University Hospital Basel, Schoenbeinstrasse 40, 4031 Basel, Switzerland

**Keywords:** COPD exacerbations, Airway remodeling, Airway obstruction, Matrix metalloproteinases, Tissue inhibitor of matrix metalloproteinases, Emphysema, Bronchoalveolar lavage

## Abstract

**Background:**

Acute exacerbations of chronic obstructive pulmonary disease (AE-COPD) are associated with accelerated aggravation of clinical symptoms and deterioration of pulmonary function. The mechanisms by which exacerbations may contribute to airway remodeling and declined lung function are poorly understood. In this study, we investigated if AE-COPD are associated with differential expression of matrix metalloproteinases (MMPs) and their tissue inhibitors (TIMPs) in bronchoalveolar lavage (BAL).

**Methods:**

COPD patients undergoing diagnostic bronchoscopy, with either stable disease (*n* = 53) or AE-COPD (*n* = 44), matched for their demographics and lung function parameters were included in this study. Protein levels of MMP-2,–9,–12 and of TIMP-1 and -2 in BAL were measured by ELISA. Enzymatic activity of MMP-2 and -9 was assessed by gelatin zymography.

**Results:**

We observed that MMP-9, TIMP-1 and TIMP-2 were significantly increased in BAL during AE-COPD. Furthermore, there was a significant negative correlation of MMP-9, TIMP-1 and TIMP-2 with FEV1% predicted and a significant positive correlation of TIMP-1 and TIMP-2 with RV% predicted in AE-COPD. None of MMPs and TIMPs correlated with DLCO% predicted, indicating that they are associated with airway remodeling leading to obstruction rather than emphysema. In AE-COPD the gelatinolytic activity of MMP-2 was increased and furthermore, MMP-9 activation was significantly up-regulated irrespective of lung function, bacterial or viral infections and smoking.

**Conclusions:**

The results of this study indicate that during AE-COPD increased expression of TIMP-1, TIMP-2, and MMP-9 and activation of MMP-9 may be persistent aggravating factors associated with airway remodeling and obstruction, suggesting a pathway connecting frequent exacerbations to lung function decline.

**Electronic supplementary material:**

The online version of this article (doi:10.1186/s12931-015-0240-4) contains supplementary material, which is available to authorized users.

## Background

Chronic obstructive pulmonary disease (COPD) is a chronic inflammatory respiratory condition, characterized by progressive airflow limitation that is not fully reversible. The accelerated deterioration of pulmonary function may occur at any stage in the course of the disease. The associated episodes are called acute exacerbations of COPD (AE-COPD). Frequent AE-COPD hasten lung function decline affecting quality of life, exercise capacity and survival in patients with COPD [1].

AE-COPD can be triggered by viral or bacterial infection of the lower respiratory tract [2, 3] and stimulate both airway and systemic inflammatory responses [4]. Chronic inflammation in COPD and AE-COPD are associated with disturbances in the homeostasis of extracellular matrix (ECM) molecules, thereby contributing to airway remodeling, which is a hallmark of the disease [5, 6]. In the lung, the ECM is subjected to a daily turnover of about 10 %, indicating that subtle changes in turnover rates accumulate to large changes in total ECM composition with time.

Matrix metalloproteinases (MMPs) are proteolytic enzymes that degrade ECM components both under physiological conditions and in pathological processes. In healthy lung, MMPs and their physiological tissue inhibitors (TIMPs) are produced by various cell types and their actions are essential for many physiological processes, such as wound healing and cell trafficking [7, 8]. Apart from digesting components of the ECM, MMPs modulate the activity of other proteases and cytokines [9–11]. Deregulation of the balance between MMPs and TIMPs or inappropriate secretion of MMPs by structural or inflammatory cells are determinant factors in the pathophysiology of lung diseases, particularly COPD [12].

MMPs are divided into subclasses according to their substrate specificity and structural characteristics [13]. MMPs are produced by a range of stromal cells as well as by neutrophils and alveolar macrophages, which are the major inflammatory cells implicated in COPD [8, 14]. Previous studies have shown that the levels of MMPs are elevated in the bronchoalveolar lavage (BAL) fluid from patients with COPD, as compared to controls [14, 15] and in the sputum of COPD patients during exacerbation [16]. However, there are no data available for the expression of MMPs and TIMPs in BAL during AE-COPD. Moreover, it is uncertain how MMPs and TIMPs relate to disease severity and phenotype, such as airway remodeling and emphysema as assessed by body plethysmography and diffusion capacity, as well as by CT-scan.

In the present study, we hypothesized that the expression and/or activity of MMPs and TIMPs is modified during acute exacerbation of COPD leading to ECM remodeling associated to lung function limitation. To test this hypothesis, we investigated a well-characterized cohort of patients with stable and exacerbated COPD undergoing bronchoalveolar lavage.

## Methods

### Study subjects

This is a prospective, single center, observational study, including 97 COPD patients with stable (*n* = 53) and AE-COPD (*n* = 44) undergoing bronchoscopy at the Clinic of Respiratory Medicine, University Hospital Basel, Switzerland. All patients gave their written informed consent to participate in the study, which was performed according to Helsinki regulation and applied to the GCP guidelines and was approved by the Institutional Review Board (EKBB 295/07).

COPD was diagnosed according to the GOLD guidelines [17]. Patients were considered stable in the absence of worsening of respiratory symptoms associated with an exacerbation for at least 4 weeks before bronchoscopy. Acute exacerbation of COPD was defined as an event in the natural course of the disease characterized by a change in the patient’s baseline dyspnea, cough, and/or sputum that is beyond normal day-to-day variations, is acute in onset, and may warrant a change in regular medication in a patient with underlying COPD [17]. In patients with AE-COPD, bronchoscopies were performed between 2 and 10 days (mean of 7 days) after the onset of exacerbation. Considering that: (a) more than 50 % of patients of the AE-COPD group were COPD patients with GOLD stage III and IV; (b) 80 % of the patients in the AE-COPD group were hospitalized and (c) patients undergoing bronchoscopy at exacerbation were the ones with persistent symptoms, thus justifying the clinical indication for bronchoscopy, the exacerbations could be considered as clinically relevant severe exacerbations.

### Bronchoscopy and BAL sampling

Bronchoscopies were performed, according to standard procedures [18]. BAL was performed by three installations of 50 ml each of a pyrogenic-free, sterile, 0.9 % NaCl solution over the working channel of the bronchoscope. Recovered BAL was passed through a gauze swab, transferred into polypropylene tubes and centrifuged for 10 min at 400xg. The cell-free BAL fluid was stored at -80 °C. The cell pellet was washed twice with PBS and counted. Cytospins of about 50,000 cells were prepared using a cyto-centrifuge and stored at -20 °C.

Cytological analysis of BAL samples including differential cell count was performed [19, 20]. Microbiological analysis of BAL samples included appropriate stains, cultures and PCR for bacteria, mycobacteria, fungi and Pneumocystis jiroveci [19]. Viral infections were also investigated for rhinovirus, RSV, HSV type 1 and 2, parainfluenza 1 and 3 and CMV, by PCR, culture or immunofluorescence [19].

### Analysis of MMPs and TIMPs

Protein content in BAL fluid was determined with the standard Bradford assay (Bio-Rad, Glattbrugg, Switzerland) [21]. The gelatinolytic activity in BAL was determined in aliquots containing 2 μg of proteins using gelatin zymography [21]. Gelatinolytic activity was quantified using the computer-assisted image analysis program of Kodak (Eastman Kodak, Rochester, NY).

Activation of latent gelatinases was performed by pre-incubation with 1 mmol/L 4-aminophenylmercuric acetate (Sigma), dissolved in dimethyl sulfoxide, in 50 mmol/L Tris-hydrochloric acid, pH 7.3, containing 200 mmol/L NaCI and 5 mmol/L CaCl_2_, for 24 h at 37 °C.

Protein levels of MMP-2, MMP-9, MMP-12, TIMP-1 and TIMP-2 in BAL were measured using ELISA systems (Boster Biological Technology, Wuhan, PRC).

Cytospins of BAL were also analyzed by immunocytochemistry for the expression of MMPs using specific antibodies for MMP-2 and MMP-9 (Spring Bioscience, Pleasanton, USA) and for MMP-12, (Bioss). The staining was performed using the Bond Polymer Refine Detection kit (Leica Biosystems, Newcastle, UK). Specificity of the staining was also tested following the same incubation protocol in the absence of the antibody.

### Statistical analysis

For data analysis Statistical Package for Social Sciences (SPSS®, IBM) version 21.0 was used. To evaluate the relationship among different parameters we used a logistic multivariate regression model analysis [19].

### Power calculation

The sample size of 97 COPD patients was based on the ability to detect a difference between protein levels of MMP-9 in the BAL given a projected MMP-9 mean of 10.5 μg/g [1.2 to 21.1] at stable COPD and 17.1 μg/g [9.3 to 48.7] at exacerbated COPD (16). Assuming a standard deviation of 10 μg/g, a total of 84 patients, 42 in each group, were needed to detect a difference in MMP-9 levels between stable and AE-COPD with a power of 85 % using a two-sided *p* = 0.05 level test.

## Results

### Demographic and baseline characteristics of the patients

The cohort represented a homogenous population of adequately treated, elderly patients with a relevant smoking history, severe obstruction, markedly impairment of the diffusion capacity, mild hyperinflation, mild to moderate hypoxemia and significant emphysema.

The most common indication for bronchoscopy among patients with stable COPD was coin lesions (27 %), evaluation of bronchoscopical or surgical lung volume reduction procedures (25 %), infiltrates (16 %) and hemoptysis (8 %).

As shown in Table 1, there were no significant differences in the age, gender, number of current smokers and smoking history between patients with stable COPD and with AE-COPD. Clinical characteristics of patients with stable COPD and AE-COPD were also similar. There were no significant differences in the stage of the disease or other clinical parameters such as FEV1 % predicted, FEV1/VCmax, TLC % predicted, RV % predicted, RV/TLC % predicted, DLCO % predicted, paO_2_, paCO_2_ and HbCO % between the two groups of patients.

**Table 1:** Demographics and baseline characteristics of COPD patients

Characteristics	Stable COPD *n* = 53	AE-COPD *n* = 44	*p* value
Age, years (range)	67.9 (48-81)	69.5 (46-86)	0.402
Gender, males %	60.3	56.8	0.723
Current smokers, n (%)	15 (28.3)	13 (29.5)	0.471
Smoking history (Pack-years), mean (SD)	53.10 (23.83)	48.87 (34.1)	0.080
Severity of COPD – GOLD Stage			0.109
I (FEV_1_ ≥ 80 % predicted), n	6	2	
II (50 % predicted ≤ FEV_1_ ≤ 80 % predicted), n	20	14	
III (30 % predicted ≤ FEV_1_ ≤ 50 % predicted), n	14	21	
IV (FEV_1_ ≤ 30 % predicted), n	13	6	
VC % predicted (SD)	89.6 (21.4)	87.7 (17.8)	0.658
FEV_1_ % predicted (SD)	50.3 (21.5)	48.0 (15.9)	0.543
FEV_1_/VCmax (SD)	43.3 (13.7)	43.8 (13.7)	0.846
TLC % predicted (SD)	114.7 (22.3)	111.9 (20.0)	0.721
RV % predicted (SD)	162.8 (61.5)	157.6 (45.1)	0.290
DLCO % predicted (SD)	48.3 (19.2)	45.7 (18.3)	0.507
RV/TLC (SD)	0.55 (0.11)	0.56 (0.09)	0.988
paO_2_, kPa (SD)	8.8 (1.5)	9.2 (1.5)	0.294
paCO_2_, kPa (SD)	5.3 (0.8)	5.2 (0.8)	0.399
HbCO % (SD)	2.6 (2.5)	2.2 (2.1)	0.414
CT scan assessment, n	29	32	
Bronchiectasis	4	7	
Moderate emphysema	9	10	
Severe emphysema	17	17	
COPD-medication, n (%)			
SABA	11 (21)	10 (23)	0.768
LABA	7 (13)	5 (11)	0.816
LAMA	8 (15)	14 (32)	0.043
SABA + LAMA	10 (19)	12 (28)	0.295
LABA + ICS	37 (70)	28 (65)	0.625
ICS	3 (6)	5 (12)	0.460
oral corticosteroids	18 (34)	19 (44)	0.306
Xanthines	2 (4)	1 (2)	1.000
Mucolytics	5 (9)	11 (26)	0.035
LTOT	11 (21)	12 (28)	0.414

*n* number of patients, *SD* standard deviation, *FEV1* forced expiratory volume in one second, *RV* residual volume, *DLCO* diffusion capacity of the lung for carbon monoxide, *TLC* transfer factor of the lung for carbon monoxide, *VC* vital capacity, *SABA* short-acting β_2_-agonists, *LABA* long-acting β_2_-agonists, *LAMA* long-acting muscarinic antagonists, *ICS* inhaled corticosteroids, *LTOT* long term oxygen therapy

Medication including short-acting β_2_-agonists alone or in combination with long-acting muscarinic antagonists (LAMA), long-acting β_2_-agonists alone or in combination with inhaled corticosteroids (ICS), ICS alone, oral corticosteroids and xanthines was also similar between stable COPD and AE-COPD patients. However, significantly more AE-COPD patients received LAMA (*p* = 0.043) and mucolytics (*p* = 0.035) than stable COPD patients.

### Microbiology and cytology of BAL

Microbiological analysis of BAL samples revealed potential pathogenic bacteria in 43 cases (41.5 % in stable vs. 47.7 % in exacerbation, *p*-value = 0.404) (Table 2). In contrast to bacterial, viral detection was more common among patients with an acute exacerbation (*n* = 17, 9.4 % in stable vs 27.3 % in exacerbation, *p*-value = 0.041.) Most common viruses encountered were cytomegalovirus and respiratory syncytial virus, reflecting the severity of the disease and the common use of inhaled/oral steroids. One patient with stable COPD and 4 patients with AE-COPD had both bacterial and viral detection in the BAL. Aspergillus colonization was evidenced in 16 cases (13.2 % in stable vs 20.4 % in exacerbation).

**Table 2:** Microbiological analysis of BAL samples

	Stable COPD *n* = 53	AE-COPD *n* = 44
Bacteriology positive	22 (41.5)	21 (47.7)
n (% of patients)		
Gram staining		
Gram positive cocci	14 (26.4)	13 (29.5)
Gram positive rods	9 (17.0)	4 (9.1)
Gram negative rods	6 (11.3)	7 (15.9)
Culture		
Streptococcus pneumonia	4 (7.5)	-
Streptococcus pyogenes	-	2 (4.5)
Moraxella catarrhalis	-	1 (2.3)
Staphylococcus aureus	-	2 (4.5)
Haemophilus influenza	2 (3.8)	-
Enterobacteriace	4 (7.5)	3 (6.8)
Enterococci	-	1 (2.3)
Escherichia coli	1(1.9)	2 (4.5)
Pseudomonas aeruginosa	-	2 (4.5)
Klebsiella pneumoniae	-	1 (2.3)
Virology positive		
n (% of patients)	5 (9.4)	12 (27.3)
Rhinovirus	1 (1.9)	-
RSV	-	4 (9.1)
HSV type 1	1 (1.9)	2 (4.5)
HSV type 2	-	1 (2.3)
parainfluenza 1	1 (1.9)	-
parainfluenza 3	1 (1.9)	-
CMV	1 (1.9)	5 (11.4)
Bacteriology and virology positive	1 (1.9)	4 (9.1)
Fungi		
n (% of patients)	16 (30.2)	23 (52.3)
Aspergillus	7 (13.2)	9 (20.4)
Candida albicans	6 (11.3)	8 (18.1)
Other	3 (5.7)	6 (13.6)

*AE-COPD* acute exacerbations of COPD, *RSV* respiratory syncytial virus, *HSV* herpes simpex virus, *CMV* cytomegalovirus, *n* = number of patients

Semi-quantitative differential cell counts evidenced a pleocytosis in 44 of the cases. Additionally, quantitative differential cell count was possible in 31 samples of the stable COPD group and in 22 samples of the AE-COPD group. The percentage of macrophages, neutrophils, lymphocytes and eosinophils was not significantly different between stable COPD and AE-COPD (Table 3).

**Table 3:** Cytological analysis of BAL samples

Cell type	Stable COPD	AE-COPD	*p* value
Quantitative differential cell count	*n* = 31	*n* = 22	
% of total cells ± SEM			
macrophages	68.69 ± 5.44	57.72 ± 6.75	0.357
neutrophils	26.50 ± 5.58	33.84 ± 6.98	0.582
lymphocytes	5.08 ± 0.67	6.08 ± 1.16	0.828
eosinophils	0.26 ± 0.09	2.20 ± 1.05	0.095

*AE-COPD* acute exacerbations of COPD, *n* number of patients, *SEM* standard error of the mean

### MMPs and TIMPS are differentially expressed in the BAL of patients with AE-COPD

**Fig. 1:**
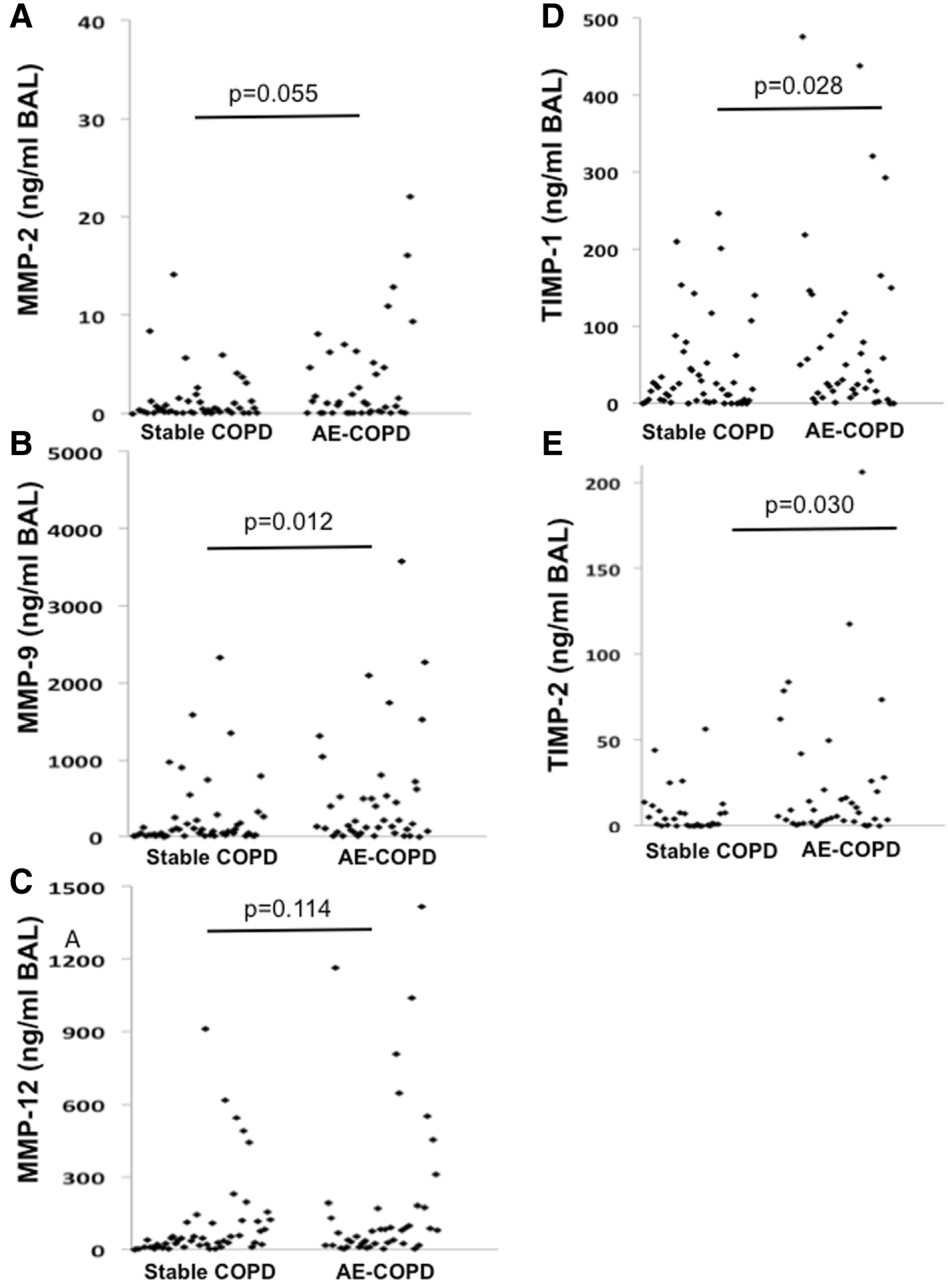
Concentration of MMPs and TIMPs in BAL of COPD patients. MMPs and TIMPs were measured in aliquots of BAL from COPD patients at a stable state and during acute exacerbations (AE-COPD) by ELISA. Figures depict representative distribution of the values between different patients. The mean and median values, the standard error of the mean and the standard deviation of these measurements are shown in Table 4

MMP-9, TIMP-1 and TIMP-2 were significantly increased in AE-COPD (Fig. 1a–e, Table 4). However, there were no significant differences in the molar ratios of MMP-2/TIMP-2 and MMP-9/TIMP-1 between AE-COPD and stable COPD.

**Table 4:** Descriptive statistics for the concentration of MMPs and TIMPs in BAL of COPD patients

Parameter	COPDstatus	Mean	SEM	SDEV	Median	Min	Max	*p* value
MMP-2 (ng/ml BAL)	AE	4.34	1.41	8.68	1.16	0.01	48.44	0.055
	stable	3.25	2.06	13.50	0.36	0.01	88.33	
MMP-9 (ng/ml BAL)	AE	670.13	193.04	1,189.97	206.55	0.01	6,158.33	0.012
	stable	284.51	74.49	488.48	88.59	0.80	2,331.00	
MMP-12 (ng/ml BAL)	AE	209.44	56.39	347.64	79.37	0.52	1,415.38	0.114
	stable	111.73	28.39	186.17	44.53	2.57	910.00	
TIMP-1 (ng/ml BAL)	AE	86.77	19.21	118.43	35.66	0.01	476.25	0.028
	stable	49.23	9.59	62.88	25.00	0.01	246.15	
TIMP-2 (ng/ml BAL)	AE	22.69	6.60	40.70	5.47	0.01	206.04	0.030
	stable	6.23	1.33	8.71	2.67	0.01	43.64	
MMP-2/TIMP-2 (molar ratio)	AE	0.85	0.38	2.37	0.078	0.00	12.90	0.791
	stable	2.66	2.05	13.43	0.081	0.00	88.32	
MMP-9/TIMP-1 (molar ratio)	AE	12.01	2.84	17.49	5.18	0.00	71.48	0.284
	stable	13.88	4.09	26.81	4.45	0.06	135.30	

*AE* acute exacerbation, *SEM* standard error of the mean, *SDEV* standard deviation, Min lower value, Max higher value, *MMP* matrix metalloproteinase, *TIMP* tissue inhibitor of MMP

Bacterial or viral infections were not associated with significant changes in the levels of MMPs and TIMPs in the BAL of patients with stable or with AE-COPD (Additional files 1, 2, 3 and 4).

**Fig. 2:**
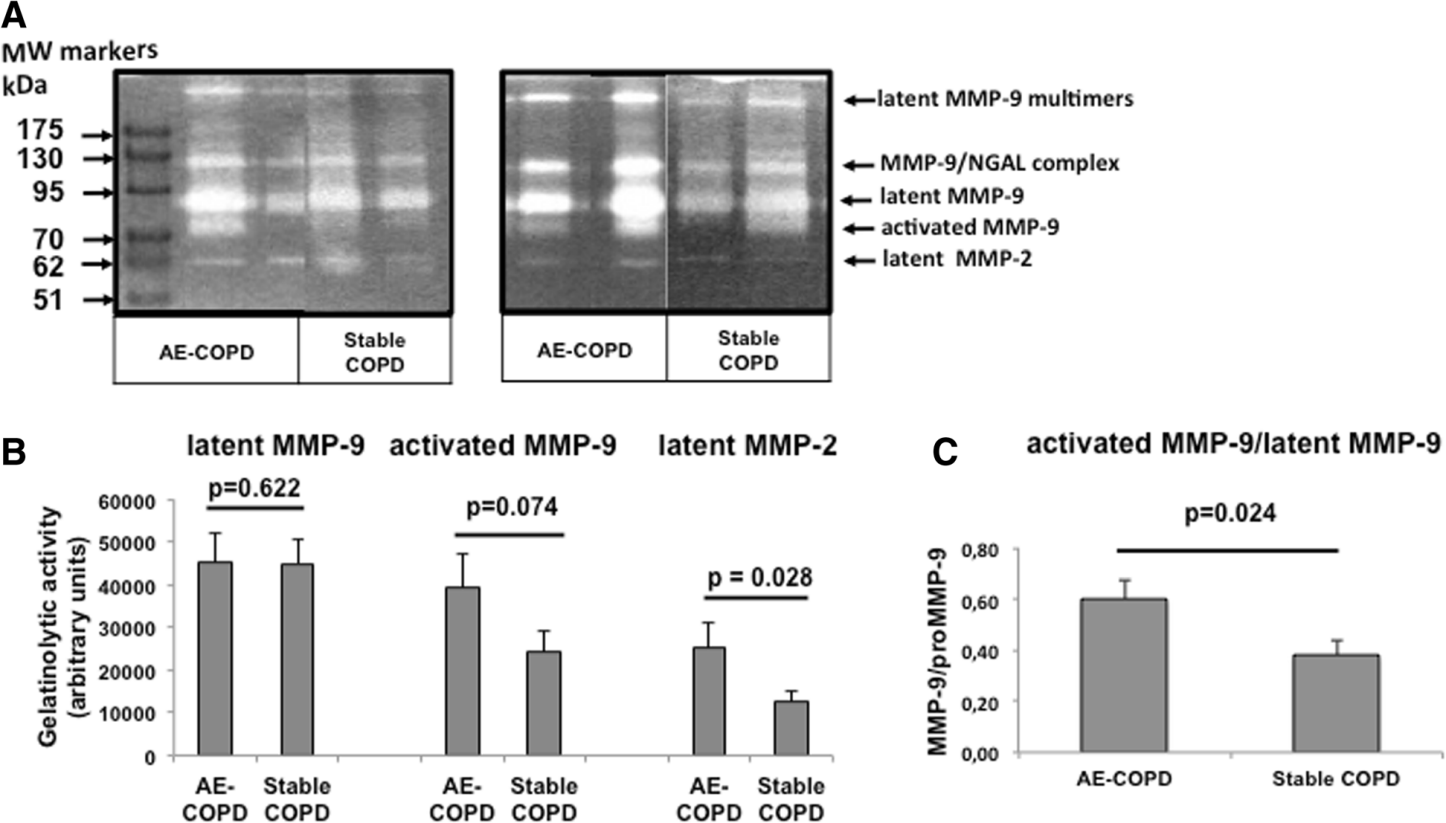
Gelatin zymography analysis. **a** Representative gelatin zymography of BAL aliquots (2 μg of protein) from 4 patients with AE-COPD and 4 patients with stable COPD. Bands of enzymatic activity were visualized by negative staining with standard Coomassie brilliant blue dye solution. **b** Quantitative analysis of gelatinase activity from zymograms using a computer-supported image analysis program. Values are mean ± SEM of zymograms from BAL samples obtained from 54 patients with stable COPD and 43 patients with AE-COPD. **c** Ratio of activated MMP-9 to latent MMP-9

Gelatin zymography analysis revealed several bands of gelatinolytic activity (Fig. 2a). The 200 kDa band corresponds to multimers of latent MMP-9. The band of 130 kDa corresponds to MMP-9 in complex with lipocalin (NGAL), indicating that neutrophils are a significant source of MMP-9. The bands of 92 kDa and 83 kDa correspond to latent MMP-9 and activated MMP-9, respectively. The band of 64 kDa corresponds to latent MMP-2. The identity of these bands was also confirmed after treatment of BAL samples with 4-aminophenylmercuric acetate, which activates MMPs (data not shown).

Quantitative analysis of the lysis bands revealed that there were no significant differences in the gelatinolytic activity of latent MMP-9 and activated MMP-9 in BAL between patients with stable COPD and AE-COPD (Fig. 2b). However, the gelatinolytic activity of latent MMP-2 was significantly increased in the BAL of AE-COPD patients (Fig. 2b). Furthermore, the ratio of activated MMP-9/latent MMP-9 was significant higher in AE-COPD, indicating increased activation of MMP-9 in AE-COPD (Fig. 2c). Overall, the incidence of latent MMP-9 and activated MMP-9 forms was similar in stable and exacerbated COPD (43.4 % in stable vs 42.9 % in exacerbated, *p*-value = 0.958). However, there was a significantly higher activation of MMP-9 at exacerbation, as compared to the stable state (*p*-value = 0.024).

### Cell expression of MMPs

**Fig. 3:**
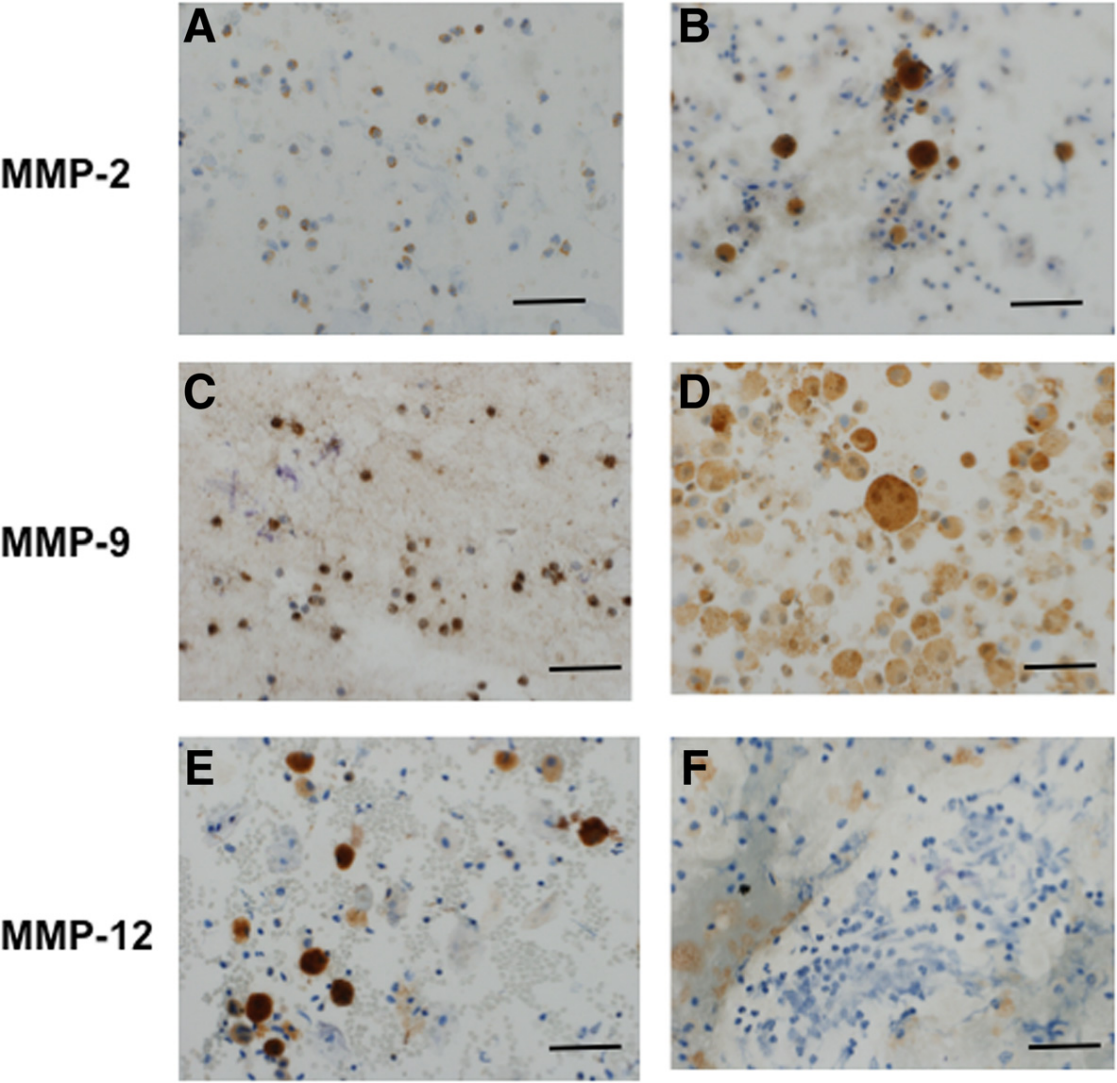
Immunocytochemistry. Immunocytochemical detection of MMP-2, MMP-9 and MMP-12 protein expression on cytospins of BAL from patients with COPD (magnification 200x). MMP-2 and MMP-9 expression was observed in neutrophils and macrophages (**a**-**d**). MMP-12 expression was only observed in macrophages (**e**) and not in neutrophils (**f**). Insert bars represent 50 μm

Immunocytochemistry of BAL cells showed that macrophages and neutrophils stained positively for MMP-2 (Fig. 3a–b) and MMP-9 (Fig. 3c–d), respectively. However, only macrophages but not neutrophils stained positively for MMP-12 (Fig. 3e–f). The same staining pattern was observed in BAL cells from either stable COPD or AE-COPD patients.

### Spearman’s correlation analysis

We further investigated the correlation of MMPs and TIMPs with FEV1%. We observed a significant negative correlation of MMP-9 with FEV1% in the group of AE-COPD but not in the stable COPD group (Table 5, Additional file 5, C-D). Similar results were also obtained when we performed correlation analysis with FEV/FVC (Table 5). There was no correlation between MMP-9 with RV% predicted or DLCO% predicted in neither group, indicating that MMP-9 correlate with airway tissue remodeling leading to airway obstruction rather than to emphysema. MMP-2, MMP-9, MMP-12, TIMP-1 and TIMP-2 did not correlate with the age of the patients or their smoking history (Table 5).

**Table 5:** Correlation analysis of protein levels in BAL of MMPs and TIMPs (Spearman’s rho)

	Stable COPD *n* = 53		AE-COPD *n* = 44	
	rho	*p* value	rho	*p* value
Age of patients				
MMP-2	-0.162	0.266	-0.113	0.482
MMP-9	0.090	0.527	0.001	0.931
MMP-12	0.072	0.616	-0.055	0.726
TIMP-1	-0.021	0.884	-0.067	0.675
TIMP-2	0.015	0.922	-0.141	0.379
Packs per year				
MMP-2	-0.102	0.499	0.126	0.452
MMP-9	-0.236	0.103	-0.042	0.802
MMP-12	-0.149	0.317	-0.033	0.839
TIMP-1	-0.069	0.636	0.012	0.940
TIMP-2	-0.088	0.578	-0.101	0.547
FEV1 % predicted				
MMP-2	-0.140	0.339	-0.186	0.251
MMP-9	-0.241	0.085	-0.475^a^	0.002
MMP-12	-0.242	0.087	-0.281	0.071
TIMP-1	-0.162	0.246	-0.510^a^	0.001
TIMP-2	-0.166	0.281	-0.492^a^	0.001
FEV/FVC				
MMP-2	-0.049	0.741	-0.130	0.424
MMP-9	-0.151	0.284	-0.364^b^	0.019
MMP-12	-0.107	0.457	-0.180	0.254
TIMP-1	-0.114	0.418	-0.420^a^	0.006
TIMP-2	-0.177	0.250	-0.365^b^	0.020
RV % predicted				
MMP-2	-0.113	0.445	0.175	0.287
MMP-9	0.106	0.459	0.307	0.054
MMP-12	0.042	0.773	0.099	0.537
TIMP-1	0.135	0.339	0.365^b^	0.021
TIMP-2	0.161	0.302	0.344^b^	0.032
DLCO % predicted				
MMP-2	-0.109	0.475	0.097	0.566
MMP-9	-0.057	0.700	0.079	0.636
MMP-12	-0.026	0.860	0.058	0.725
TIMP-1	-0.016	0.911	-0.125	0.454
TIMP-2	-0.199	0.219	0.069	0.684

*AE* acute exacerbation, *MMP* matrix metalloproteinase, *TIMP* tissue inhibitor of MMP

^a^Correlation is significant at the 0.01 level (2-tailed)

^b^Correlation is significant at the 0.05 level (2-tailed)

We also observed a significant negative correlation between TIMP-1 and TIMP-2 with FEV1% predicted only in the AE-COPD group (Table 5, Additional file 5, G–J), indicating that both TIMPs are associated with airway obstruction in COPD. Furthermore, both TIMPs were positively correlated with RV% predicted in the AE-COPD group, indicating that both TIMPs are associated with emphysematous parenchymal destruction in AE-COPD (Table 5, Additional file 6, G–J).

### Logistic multivariate regression model

In order to balance stable and AE-COPD group concerning several influencing covariates, a one to one propensity score matching was performed. It was possible to find 35 matching subjects (Table 6).

**Table 6:** Propensity score matching

Variable	Stable COPD *n* = 35	AE-COPD *n* = 35	*P*-Value
Age	69.2 (6.44)	69.5 (9.80)	0.863
Sex	0.37 (0.49)	0.43 (0.50)	0.632
Packs per year	50.6 (23.1)	47.9 (36.1)	0.715
FEV1 % predicted	50.7 (21.6)	48.6 (16.3)	0.653
RV % predicted	153 (46.5)	160 (45.9)	0.527
DLCO % predicted	46.8 (19.8)	46.2 (16.7)	0.885
Bacteriology			1.000
negative	19 (54.3 %)	19 (54.3 %)	
positive	16 (45.7 %)	16 (45.7 %)	
Virology			0.511
negative	31 (88.6 %)	28 (80.0 %)	
positive	4 (11.4 %)	7 (20.0 %)	
activation of MMP^a^	1.28 (0.97)	0.56 (0.56)	0.018

*AE* acute exacerbation, *FEV1* forced expiratory volume in one second, *RV* residual volume, *DLCO* diffusion capacity of the lung for carbon monoxide

^a^Expressed as the ratio of proMMP-9/MMP-9

Additionally a logistic regression predicting exacerbation from the ratio of activated MMP-9/latent MMP-9 was performed with the matched dataset. This ensures that the prediction is balanced conditional on the matching. The ratio of activated MMP-9/latent MMP-9, which indicates the activation of MMP-9 (functional enzyme), was significantly associated with AE-COPD and this association was independent from demographic characteristics of the patients, smoking status, lung function and infections (Table 7).

**Table 7:** Logistic, multivariate regression model for the prediction of AE-COPD among the study population

Variable	OR 95 %-CI	*p* value
activation of MMP-9	0.159 (0.031-0.816)	0.028
Age	1.064 (0.958-1.181)	0.244
Sex	2.535 (0.189-33.935)	0.482
Packs per year	0.978 (0.935-1.022)	0.320
FEV1 % predicted	1.027 (0.947-1.115)	0.513
RV % predicted	1.003 (0.974-1.033)	0.859
DLCO % predicted	0.952 (0.870-1.042)	0.286
Bacteriology	0.361 (0.043-3.055)	0.350
Virology	0.264 (0.014-4.919)	0.372

*AE* acute exacerbation, *FEV1* forced expiratory volume in one second, *RV* residual volume, *DLCO* diffusion capacity of the lung for carbon monoxide

## Discussion

In this well-characterized cohort, including well-matched stable and exacerbated patients with COPD, we demonstrated for the first time significant alterations in ECM proteins during acute exacerbation, as compared to the stable state in BAL. We have found a significant increase of MMP-9, TIMP-1 and TIMP-2 protein levels at exacerbation. While MMP-9, TIMP-1 and TIMP-2 were negatively associated with FEV_1_% predicted, TIMP-1 and TIMP-2 were positively associated with residual volume. Therefore, MMP-9 and TIMPs seem to be associated with airway obstruction while TIMPs are potentially related to tissue destruction respectively emphysema. Furthermore, MMP-9 is markedly activated at AE-COPD, as compared to the stable state, and this activation is independent of clinical characteristics including age, gender, cigarette consumption, severity of obstruction and emphysema as well as infectious agents at exacerbation. Taken together, these findings suggest that MMP-9 and TIMPs may play a significant role in airway remodeling and subsequent lung function decline following AE-COPD.

It has been previously reported that there is an overexpression of MMP-9 in bronchiectatic airways in vivo [22] and that MMP-9 levels are increased in exhaled breath condensate in children with bronchiectasis [23]. In our study, we included 4 patients with stable COPD and 7 patients with AE-COPD that were also diagnosed with bronchiectasis. In order to investigate if inclusion of these patients may affect our results, we repeated measurements of MMP-9 following exclusion of patients with bronchiectasis from both groups. We obtained very similar results since we found that MMP-9 was significantly increased in the BAL of AE-COPD patients as compared to stable COPD (*p* = 0.027), indicating inclusion of this small number of patients with bronchiectasis in both groups did not influence our results.

Furthermore, we investigated if treatment of COPD patients with oral steroids may affect the levels of MMP-9 in BAL. We found that there was a significant increase (*p* = 0.019) in the levels of MMP-9 in COPD patients that received oral steroids (483 ng/ml ± 108), as compared to COPD patients that did not receive oral steroids (402 ng/ml ± 132). These results were expected since COPD patients that receive oral steroids are patients with more severe disease and there is a significant negative correlation of MMP-9 with FEV1% predicted, reflecting disease severity. Nevertheless, since the number of patients that received oral steroids is the same in the group of stable COPD and in the group of AE-COPD we believe that the use of oral steroids did not influence the results and conclusions of our study.

Previous data suggest that bacterial infections could induce the expression [24] or the activity [25] of MMPs. However, in the current study, neither bacterial nor viral infections were associated with significant changes in the levels of MMPs and TIMPs in BAL, indicating that the observed increase of MMP-9, TIMP-1 and TIMP-2 in BAL during AE-COPD resulted from increased secretion by lung resident cell types or by inflammatory cells such as macrophages and neutrophils invading the lung during AE-COPD. Interestingly, the number of macrophages and neutrophils, which are the main cell types producing MMPs and TIMPs, were similar in both groups of patients. Therefore, it is tempting to hypothesize that the observed differences in MMPs and TIMPs in AE-COPD resulted either from a different cell source, such as alveolar type II cells, bronchial epithelial cells, Clara cells, fibroblasts or smooth muscle cells, or from induced secretion of MMPs and TIMPs by macrophages and neutrophils during AE-COPD.

It has been shown that MMP-9 and TIMP-1 levels are increased in exhaled breath condensate of AE-COPD patients [26] and that MMP-9 levels are increased in sputum of AE-COPD patients [16]. In our study, we showed that TIMP-1, as well as TIMP-2 levels were significantly increased in BAL during AE-COPD and that the molar ratios of MMP-9/TIMP-1 and MMP-2/TIMP-2 were not altered significantly. TIMPs bind to MMPs in 1:1 molar ratio to inhibit their activity. However, MMPs do not only act as secreted enzymes, but they also bind to cell surface where they exert elastolytic activity. Approximately 80 % of MMP-9, synthesized by neutrophils, remains associated with the surface of the cells and is not neutralized by TIMPs [27]. Furthermore, in different parts of the microenvironment of the airways, MMPs or TIMPs may predominate and therefore, the increased levels of TIMPs in BAL during AE-COPD may not necessarily result in inhibition of MMP-9 activity.

MMP-2 activity was also increased in BAL during AE-COPD, which is in good agreement with previous studies showing that there is increased immune-reactivity for MMP-2 in the lungs of patients with COPD, mainly in alveolar macrophages and airway epithelial cells [28] and that there is increased expression and activity of MMP-2 in the lungs and sputum of patients with COPD [29].

Despite the available literature on the role of MMPs and TIMPs in COPD, the information regarding the involvement of these molecules in AE-COPD remains elusive. In our study, we report that there is a significant increase in the concentration of MMP-9 in BAL during AE-COPD and moreover, a significant activation of the enzyme, independent of age, sex, smoking status, lung function parameters and microbiology. These results indicate that activation of MMP-9 is a parameter directly associated with AE-COPD irrespective of demographic or clinical characteristics of the COPD patients.

It has been shown that in patients with emphysema, there is elevated concentration of MMP-1 and MMP-9 in BAL and increased expression of these MMPs in macrophages [8]. Alveolar macrophages from healthy smokers express more MMP-9 than those from healthy non-smokers [30] and MMP-9 expression is increased in cells from patients with COPD [31], with enhanced elastolytic activity [32]. MMP-9 and the ratio of MMP-9 to TIMP-1 are increased in induced sputum of patients with COPD [33]. MMP-9 activity is increased in the lung parenchyma of patients with emphysema [34], and correlated with FEV1 [35]. Here, we observed a significant negative correlation of MMP-9, TIMP-1 and TIMP-2 with FEV1% predicted and of TIMP-1 and TIMP-2 with RV% predicted in the AE-COPD group. The fact that none of the investigated molecules correlated with DLCO% predicted indicates that they are associated with tissue remodeling leading to airway obstruction but not to emphysema.

It remains to be elucidated if the observed alterations in the levels and/or activity of MMPs and TIMPs that we observe in the present study are causative for AE-COPD or a consequence of the disease. Since bacterial and viral infections, which are the main causative factors for AE-COPD, were not associated with significant changes in the levels of MMPs and TIMPs in BAL, it is more likely that deregulation of these molecules are independent causative factors for AE-COPD. As MMPs and TIMPs might be casually involved in the development of an exacerbation, targeting TIMPs or gelatinases with specific and non toxic agents, such as the third generation of highly selective MMP inhibitors [36] or monoclonal antibodies against MMPs [37] may offer valuable treatments to confront airway remodeling associated with AE-COPD.

Increasing evidence suggest that MMP-12, plays a role in lung destruction in COPD. Data obtained from a large multiple cohort association study, linked the variation of MMP-12 with lung function in smokers and the risk of developing COPD [38], while, in animal models of COPD, the deletion of MMP-12 in mice exposed to cigarette smoke protects these animals from emphysema [39]. Patients with COPD have elevated levels of MMP-12 in their airways and increased numbers of MMP-12 expressing macrophages [40]. In our study, MMP-12 was increased in AE-COPD, as compared to stable COPD. However, this result was not statistically significant. Furthermore, in our cohort of AE-COPD patients we did not observe any statistically significant correlation of MMP-12 with smoking status, FEV1% predicted, RV% predicted, or DLCO% predicted.

Our study has a few limitations. It is a single center study that included adequate patients requiring diagnostic bronchoscopy. The time lapse between the onset of AE-COPD and bronchoscopy varied between patients from 2–10 days (mean of 7 days). However, when we stratified the measurements for MMP-2, MMP-9, MMP-12, TIMP-1 and TIMP-2 in BAL by the time lapse from the onset of the exacerbation (1–3, 4–6 and 7–10 days), we did not observe significant differences in the values of central tendency for any of the parameters measured. Taking into account the results of the stratified analyses, as well as the fact that patients undergoing bronchoscopy were the ones with persistent symptoms, we carefully assume that the time lapse between the onset of exacerbation and the collection of the BAL at bronchoscopy does not preclude our interpretations on the association between MMPs and TIMPs in BAL and exacerbations.

Another limitation is that we were unable to evaluate the same patient both at exacerbation and at stable state. However, this must be regarded as an almost obligatory limitation, since it would be extremely scarce to find patients that would require diagnostic bronchoscopy both at exacerbation and at stable state within a short time interval. At the same time, it would be unethical to perform, for the shake of proper research methodology, a second BAL at a patient at exacerbation, who recently underwent BAL at stable state for diagnostic reasons. To compensate this, we have analyzed almost 100 BAL samples of patients with either exacerbated or stable disease. Difficulties in performing BAL at exacerbation are well described and might explain the scarcity of data in this setting. A further aspect to consider is that the sample size provided the study sufficient power to detect clinical relevant differences between both conditions. Although the analysis of non-paired data tends to produce larger deviations from the measures of central tendency, cohorts at stable and exacerbated condition were very well balanced. This was confirmed by the endorsing results of the multivariate analysis and the propensity score. Therefore, we believe it is fair to assume that the reported differences in the expression and/or activity of MMPs and TIMPs were rather related to the manifestation of the exacerbations per se. A further strength is the fact that we could associate protein and activity values to an extensive clinical characterization, including body plethysmography results, detailed microbiology analysis and CT-scan findings.

## Conclusions

The data presented in this study indicate that during AE-COPD increased expression of TIMP-1, TIMP-2, and MMP-9 and activation of MMP-9 may be persistent aggravating factors associated with airway remodeling and obstruction, suggesting a pathway connecting frequent exacerbations to lung function decline. MMP-9 and TIMPs could potentially represent valuable therapeutic targets to counteract remodeling and parenchymal destruction associated with recurrent exacerbations.

## Additional files

Additional file 1:
**Concentration of MMPs and TIMPs in BAL of COPD patients at a stable state (** ***n*** **= 53) with positive or negative bacteriology.**
Additional file 2:
**Concentration of MMPs and TIMPs in BAL of AE-COPD patients (** ***n*** **= 44) with positive or negative bacteriology.**
Additional file 3:
**Concentration of MMPs and TIMPs in BAL of COPD patients at a stable state (** ***n*** **= 53) with positive or negative virology.**
Additional file 4:
**Concentration of MMPs and TIMPs in BAL of AE-COPD patients (** ***n*** **= 44) with positive or negative virology.**
Additional file 5:
**Correlation of MMPs and TIMPs with FEV1 % predicted.** Representative correlation of MMP-2, MMP-9, MMP-12, TIMP-1 and TIMP-2 protein levels in BAL with FEV1% predicted. Spearman’s rho analysis is shown in Table 5. Additional file 6:
**Correlation of MMPs and TIMPs with RV % predicted.** Representative correlation of MMP-2, MMP-9, MMP-12, TIMP-1 and TIMP-2 protein levels in BAL with RV% predicted. Spearman’s rho analysis is shown in Table 5.

## Abbreviations

AE-COPD: Acute exacrebations of COPD
BAL: Bronchoalveolar lavage
COPD: Chronic obstructive pulmonary disease
MMP: Matrix metalloproteinase
TIMP: Tissue inhibitors of matrix metalloproteinases
