# Supplementary material for: Acute exacerbations of COPD are associated with significant activation of matrix metalloproteinase 9 irrespectively of airway obstruction, emphysema and infection
Source: Respir Res. 2015 Jun 28;16(1):78. doi: 10.1186/s12931-015-0240-4 (PMC4531832; doi:10.1186/s12931-015-0240-4)
Supplement: Additional file 1: — Concentration of MMPs and TIMPs in BAL of COPD patients at a stable state ( n = 53) with positive or negative bacteriology. [file 12931_2015_240_MOESM1_ESM.docx]

**Additional File 1**

**Concentration of MMPs and TIMPs in BAL of COPD patients at a stable state (n=53)**

| **Parameter** | **Bacteriology** | **Mean** | **SEM** | **SDEV** | **Min** | **Max** | **P value** |
| --- | --- | --- | --- | --- | --- | --- | --- |
| MMP-2  (ng/ml BAL) | Negative | 1.12 | 0.34 | 1.88 | 0.00 | 8.39 | 0.604 |
|  | Positive | 1.64 | 0.90 | 3.62 | 0.01 | 14.08 |  |
| MMP-9  (ng/ml BAL) | Negative | 491.76 | 240.09 | 1,336.79 | 0.01 | 7,122.00 | 0.569 |
|  | Positive | 148.83 | 50.39 | 219.62 | 2.72 | 893.75 |  |
| MMP-12  (ng/ml BAL) | Negative | 133.82 | 37.46 | 208.59 | 0.24 | 910.00 | 0.062 |
|  | Positive | 30.03 | 6.57 | 27.86 | 0.00 | 113.64 |  |
| TIMP-1  (ng/ml BAL) | Negative | 44.47 | 11.53 | 64.21 | 0.01 | 246.15 | 0.847 |
|  | Positive | 37.78 | 12.24 | 54.72 | 0.00 | 209.80 |  |
| TIMP-2  (ng/ml BAL) | Negative | 8.35 | 2.68 | 13.94 | 0.01 | 55.95 | 0.217 |
|  | Positive | 6.55 | 1.39 | 5.38 | 0.01 | 17.98 |  |
| MMP-2/TIMP-2  (molar ratio) | Negative | 0.72 | 0.27 | 1.41 | 0.00 | 5.96 | 0.948 |
|  | Positive | 0.23 | 0.07 | 0.26 | 0.00 | 0.78 |  |
| MMP-9/TIMP-1  (molar ratio) | Negative | 240.15 | 229.43 | 1,277.39 | 0.03 | 7,122.00 | 0.727 |
|  | Positive | 7.73 | 2.20 | 9.58 | 0.40 | 38.08 |  |

Abbreviations: SEM: standard error of the mean; SDEV: standard deviation; Min: lower value; Max: higher value; MMP: matrix metalloproteinase; TIMP: tissue inhibitor of MMP.
