# Supplementary material for: Acute exacerbations of COPD are associated with significant activation of matrix metalloproteinase 9 irrespectively of airway obstruction, emphysema and infection
Source: Respir Res. 2015 Jun 28;16(1):78. doi: 10.1186/s12931-015-0240-4 (PMC4531832; doi:10.1186/s12931-015-0240-4)
Supplement: Additional file 2: — Concentration of MMPs and TIMPs in BAL of AE-COPD patients ( n = 44) with positive or negative bacteriology. [file 12931_2015_240_MOESM2_ESM.docx]

**Additional File 2**

**Concentration of MMPs and TIMPs in BAL of AE-COPD patients (n=44)**

| **Parameter** | **Bacteriology** | **Mean** | **SEM** | **SDEV** | **Min** | **Max** | **P value** |
| --- | --- | --- | --- | --- | --- | --- | --- |
| MMP-2  (ng/ml BAL) | Negative | 6.11 | 2.39 | 11,19 | 0.01 | 48.45 | 0.345 |
|  | Positive | 2.49 | 0.75 | 3.27 | 0.01 | 10.90 |  |
| MMP-9  (ng/ml BAL) | Negative | 415.57 | 120.69 | 566.12 | 0.01 | 2,268.00 | 0.465 |
|  | Positive | 901.05 | 344.18 | 1,539.22 | 7.96 | 6,158.33 |  |
| MMP-12  (ng/ml BAL) | Negative | 165.75 | 64.35 | 308.59 | 0.52 | 1,415.38 | 0.495 |
|  | Positive | 232.48 | 81.68 | 365.30 | 5.40 | 1,162.00 |  |
| TIMP-1  (ng/ml BAL) | Negative | 58.80 | 17.09 | 80.14 | 0.01 | 292.50 | 0.137 |
|  | Positive | 105.46 | 31.61 | 141.35 | 0,53 | 476.25 |  |
| TIMP-2  (ng/ml BAL) | Negative | 14.49 | 4.87 | 22.85 | 0.01 | 78.01 | 0.094 |
|  | Positive | 32.97 | 12.10 | 52.74 | 0.29 | 206.04 |  |
| MMP-2/TIMP-2  (molar ratio) | Negative | 1.43 | 0.68 | 3.09 | 0.00 | 12.90 | 0.054 |
|  | Positive | 0.14 | 0.06 | 0.26 | 0.00 | 1.06 |  |
| MMP-9/TIMP-1  (molar ratio) | Negative | 83.12 | 71.89 | 309.43 | 0.00 | 1,519.00 | 0.715 |
|  | Positive | 11.86 | 3.90 | 17.45 | 0.69 | 71.48 |  |

Abbreviations: AE: acute exacerbation; SEM: standard error of the mean; SDEV: standard deviation; Min: lower value; Max: higher value; MMP: matrix metalloproteinase; TIMP: tissue inhibitor of MMP.
