# Supplementary material for: Acute exacerbations of COPD are associated with significant activation of matrix metalloproteinase 9 irrespectively of airway obstruction, emphysema and infection
Source: Respir Res. 2015 Jun 28;16(1):78. doi: 10.1186/s12931-015-0240-4 (PMC4531832; doi:10.1186/s12931-015-0240-4)
Supplement: Additional file 3: — Concentration of MMPs and TIMPs in BAL of COPD patients at a stable state ( n = 53) with positive or negative virology. [file 12931_2015_240_MOESM3_ESM.docx]

**Additional File 3**

**Concentration of MMPs and TIMPs in BAL of COPD patients at a stable state (n=53)**

| **Parameter** | **Virology** | **Mean** | **SEM** | **SDEV** | **Min** | **Max** | **P value** |
| --- | --- | --- | --- | --- | --- | --- | --- |
| MMP-2  (ng/ml BAL) | Negative | 1.05 | 0.28 | 1.81 | 0.00 | 8.39 | 0.272 |
|  | Positive | 3.91 | 3.39 | 6.78 | 0.29 | 14.08 |  |
| MMP-9  (ng/ml BAL) | Negative | 367.02 | 163.34 | 1,107.81 | 0.80 | 7,122.00 | 0.692 |
|  | Positive | 297.44 | 227.19 | 454.38 | 0.01 | 974.70 |  |
| MMP-12  (ng/ml BAL) | Negative | 101.04 | 26.81 | 179.87 | 0.01 | 910.00 | 0.792 |
|  | Positive | 35.52 | 12.16 | 24.32 | 0.24 | 55.00 |  |
| TIMP-1  (ng/ml BAL) | Negative | 39.71 | 8.72 | 59.80 | 0.00 | 246.15 | 0.367 |
|  | Positive | 66.89 | 34.11 | 68.22 | 0.30 | 153.19 |  |
| TIMP-2  (ng/ml BAL) | Negative | 7.43 | 1.90 | 11.84 | 0.01 | 55.95 | 0.166 |
|  | Positive | 11.31 | 4.53 | 7.84 | 2.67 | 17.98 |  |
| MMP-2/TIMP-2  (molar ratio) | Negative | 0.56 | 0.19 | 1.19 | 0.00 | 5.96 | 0.483 |
|  | Positive | 0.32 | 0.23 | 0.40 | 0.06 | 0.78 |  |
| MMP-9/TIMP-1  (molar ratio) | Negative | 164.68 | 154.63 | 1,048.75 | 0.06 | 7,122.00 | 0.339 |
|  | Positive | 4.03 | 2.54 | 5.09 | 0.03 | 11.11 |  |

Abbreviations: SEM: standard error of the mean; SDEV: standard deviation; Min: lower value; Max: higher value; MMP: matrix metalloproteinase; TIMP: tissue inhibitor of MMP.
