# Supplementary material for: Acute exacerbations of COPD are associated with significant activation of matrix metalloproteinase 9 irrespectively of airway obstruction, emphysema and infection
Source: Respir Res. 2015 Jun 28;16(1):78. doi: 10.1186/s12931-015-0240-4 (PMC4531832; doi:10.1186/s12931-015-0240-4)
Supplement: Additional file 4: — Concentration of MMPs and TIMPs in BAL of AE-COPD patients ( n = 44) with positive or negative virology. [file 12931_2015_240_MOESM4_ESM.docx]

**Additional File 4**

**Concentration of MMPs and TIMPs in BAL of AE-COPD patients (n=44)**

| **Parameter** | **Virology** | **Mean** | **SEM** | **SDEV** | **Min** | **Max** | **P value** |
| --- | --- | --- | --- | --- | --- | --- | --- |
| MMP-2  (ng/ml BAL) | Negative | 5.18 | 1.69 | 9.55 | 0.01 | 48.45 | 0.865 |
|  | Positive | 1.78 | 0.77 | 2.31 | 0.02 | 7.02 |  |
| MMP-9  (ng/ml BAL) | Negative | 717.54 | 226.19 | 1,279.54 | 0.01 | 6,158.00 | 0.942 |
|  | Positive | 420.22 | 175.93 | 556.39 | 38.70 | 1,746.67 |  |
| MMP-12  (ng/ml BAL) | Negative | 223.59 | 63.35 | 363.94 | 0.52 | 1,415.38 | 0.487 |
|  | Positive | 108.32 | 61.25 | 193.71 | 9.87 | 648.00 |  |
| TIMP-1  (ng/ml BAL) | Negative | 86.52 | 22.40 | 126.70 | 0.01 | 476.25 | 0.494 |
|  | Positive | 63.41 | 19.89 | 62.90 | 12.63 | 218.00 |  |
| TIMP-2  (ng/ml BAL) | Negative | 22.59 | 7.33 | 40.82 | 0.01 | 206.04 | 1.000 |
|  | Positive | 24.48 | 12.69 | 40.13 | 0.01 | 117.41 |  |
| MMP-2/TIMP-2  (molar ratio) | Negative | 0.81 | 0.44 | 2.39 | 0.00 | 12.90 | 0.831 |
|  | Positive | 0.93 | 0.77 | 2.31 | 0.00 | 7.02 |  |
| MMP-9/TIMP-1  (molar ratio) | Negative | 61.53 | 48.70 | 271.15 | 0.00 | 1,519.00 | 0.709 |
|  | Positive | 7.51 | 2.46 | 7.79 | 0.69 | 27.08 |  |

Abbreviations: AE: acute exacerbation; SEM: standard error of the mean; SDEV: standard deviation; Min: lower value; Max: higher value; MMP: matrix metalloproteinase; TIMP: tissue inhibitor of MMP.
