# Supplementary figures and images for: Acute exacerbations of COPD are associated with significant activation of matrix metalloproteinase 9 irrespectively of airway obstruction, emphysema and infection
Source: Respir Res. 2015 Jun 28;16(1):78. doi: 10.1186/s12931-015-0240-4 (PMC4531832; doi:10.1186/s12931-015-0240-4)

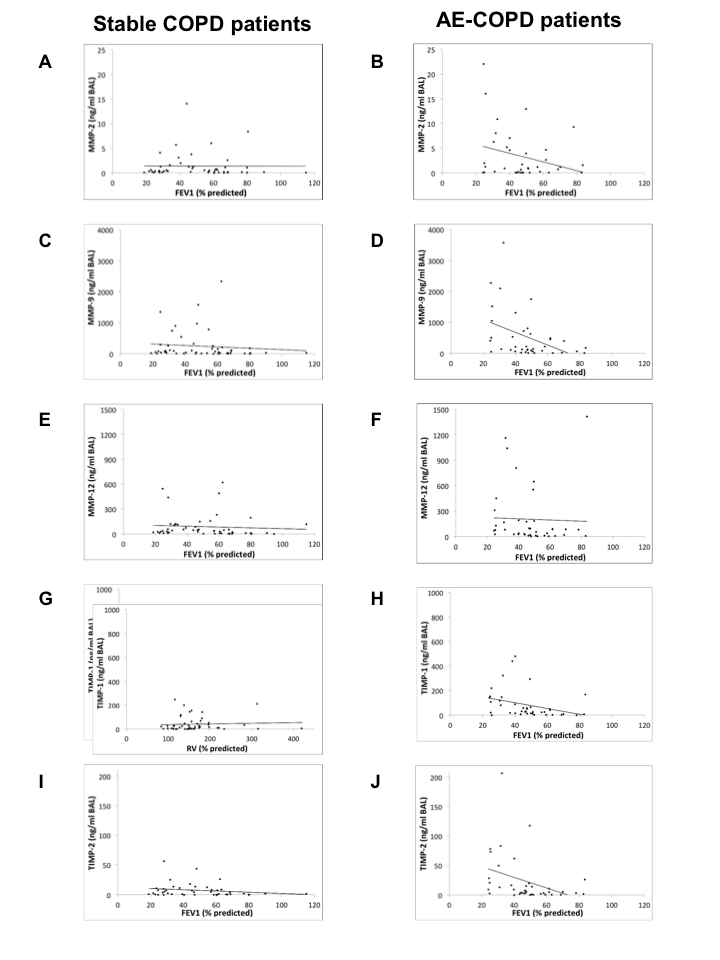

Supplement: Additional file 5: — Correlation of MMPs and TIMPs with FEV1 % predicted. Representative correlation of MMP-2, MMP-9, MMP-12, TIMP-1 and TIMP-2 protein levels in BAL with FEV1% predicted. Spearman’s rho analysis is shown in Table 5. [file 12931_2015_240_MOESM5_ESM.tiff]

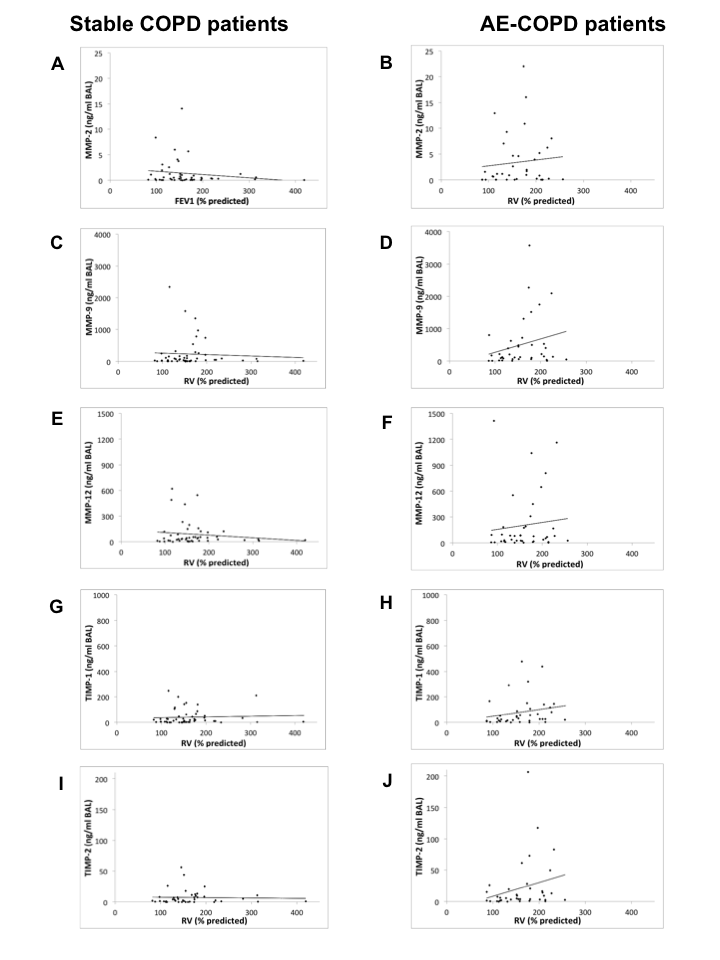

Supplement: Additional file 6: — Correlation of MMPs and TIMPs with RV % predicted. Representative correlation of MMP-2, MMP-9, MMP-12, TIMP-1 and TIMP-2 protein levels in BAL with RV% predicted. Spearman’s rho analysis is shown in Table 5. [file 12931_2015_240_MOESM6_ESM.tiff]
